# Targeted Capture of Phylogenetically Informative Ves SINE Insertions in Genus *Myotis*

**DOI:** 10.1093/gbe/evv099

**Published:** 2015-05-25

**Authors:** Roy N. Platt, Yuhua Zhang, David J. Witherspoon, Jinchuan Xing, Alexander Suh, Megan S. Keith, Lynn B. Jorde, Richard D. Stevens, David A. Ray

**Affiliations:** ^1^Department of Biochemistry, Molecular Biology, Entomology and Plant Pathology, Mississippi State University; ^2^Bionomics Research & Technology Center, Environmental and Occupational Health Science Institute, Rutgers, The State University of New Jersey; ^3^Department of Human Genetics, University of Utah Health Sciences Center; ^4^Department of Genetics, Human Genetics Institute of New Jersey, Rutgers, The State University of New Jersey; ^5^Department of Evolutionary Biology, Uppsala University, Sweden; ^6^Department of Natural Resources Management and the Museum of Texas Tech University; ^7^Department of Biological Sciences, Texas Tech University

**Keywords:** rare genomic events, Dollo parsimony, retrotransposon, phylogenetics, *Myotis lucifugus*

## Abstract

Identification of retrotransposon insertions in nonmodel taxa can be technically challenging and costly. This has inhibited progress in understanding retrotransposon insertion dynamics outside of a few well-studied species. To address this problem, we have extended a retrotransposon-based capture and sequence method (ME-Scan [mobile element scanning]) to identify insertions belonging to the Ves family of short interspersed elements (SINEs) across seven species of the bat genus *Myotis.* We identified between 120,000 and 143,000 SINE insertions in six taxa lacking a draft genome by comparing to the *M. lucifugus* reference genome. On average, each Ves insertion was sequenced to 129.6 × coverage. When mapped back to the *M. lucifugus* reference genome, all insertions were confidently assigned within a 10-bp window. Polymorphic Ves insertions were identified in each taxon based on their mapped locations. Using cross-species comparisons and the identified insertion positions, a presence–absence matrix was created for approximately 796,000 insertions. Dollo parsimony analysis of more than 85,000 phylogenetically informative insertions recovered strongly supported, monophyletic clades that correspond with the biogeography of each taxa. This phylogeny is similar to previously published mitochondrial phylogenies, with the exception of the placement of *M. vivesi.* These results support the utility of our variation on ME-Scan to identify polymorphic retrotransposon insertions in taxa without a reference genome and for large-scale retrotransposon-based phylogenetics.

## Background

Transposable elements (TEs) are repetitive sequences in eukaryotic genomes that can mobilize and/or replicate themselves. Although values vary greatly among taxa, significant portions of most eukaryotic genomes are derived from TEs. For example, approximately 85% of the corn (*Zea mays*) genome is derived from TEs (Tenaillon et al. 2011), whereas TEs compose less than 3% of the pufferfish (*Takifugu rubripes*) genome (Aparicio et al. 2002). In mammals, estimates of TE content range from approximately 40–60% (Lander et al. 2001; Waterston et al. 2002; Gentles et al. 2007; de Koning et al. 2011). By comparison, protein-coding regions make up less than 2% of the human genome (Lander et al. 2001).

TEs can be subdivided into two major classes based on their mobilization mechanism. Class I TEs (retrotransposons) mobilize through a copy and paste mechanism in which the parent retrotransposon is transcribed to RNA before being reintegrated into the genome at another location through reverse transcription. Retrotransposons are the dominant TE class in mammalian genomes and include LINEs (long interspersed elements), SINEs (short interspersed elements), LTRs (long terminal repeat), and ERVs (endogenous retroviruses). Class II TEs are referred to as DNA transposons and include a wide array of superfamilies including Tc1/Mariners, hATs, and piggyBacs. Helitrons and Mavericks are also included in this class but utilize distinct mobilization mechanisms. With the exception of the bat family Vespertilionidae, which includes the genus *Myotis*, Class II TEs have been essentially inactive in mammal genomes for the last approximately 40 Myr (Pritham and Feschotte 2007; Ray et al. 2007, 2008; Thomas et al. 2011; Pagán et al. 2012; Platt et al. 2014).

Retrotransposon insertions have been used as markers to differentiate individuals (Wang et al. 2006), populations (Ray et al. 2005; Witherspoon et al. 2006, 2013), species (López-Giráldez et al. 2005), and even larger taxonomic groups beyond the family level (Nikaido et al. 2007; Churakov et al. 2010). SINEs in particular are well suited for this purpose (Okada 1991; Hillis 1999; Ray et al. 2006). These nonautonomous retrotransposons are small, 100–500 bp (base pairs), and reach copy numbers ranging from several thousand to over 1 million in mammal genomes. Once inserted, SINEs are rarely excised (van de Lagemaat et al. 2005; Ray et al. 2006) and will be vertically inherited, becoming shared derived characters. Further, the absence of a SINE insertion at any particular locus can be safely assumed to represent the ancestral condition. The ability to characterize ancestral versus derived states makes SINE insertions valuable markers for assigning ancestry (Okada et al. 2004).

Identifying interspecific polymorphic SINE insertions in clades of nonmodel taxa has been difficult. Historically, a reference genome would be mined computationally for recent SINE insertions. Primers would be developed in the regions flanking the insertion within the reference genome and used to amplify the SINE insertion locus in other taxa. Amplicons from these reactions would be electrophoresed and scored based on band size, with SINE-containing loci (filled sites) yielding larger bands than those lacking the SINE insertion (empty sites). Although this method is effective at identifying SINE polymorphisms in the genome reference, it has limited use for identifying polymorphic insertions in taxa lacking a reference genome. In addition, large numbers of individual insertions must be assayed to identify the relatively few that are polymorphic among taxa. Recently developed sequencing technologies, laboratory methods, and the increasing availability of reference genomes provide a framework for addressing these shortcomings (Xing et al. 2013).

In particular, mobile element scanning (ME-Scan), a technique developed to identify *Alu* SINE insertions in the human genome (Witherspoon et al. 2010, 2013), has the potential to solve many of the problems associated with traditional methods of retrotransposon identification. ME-Scan utilizes a combination of computational and molecular techniques to build Illumina sequencing libraries that are enriched for the targeted SINEs. Although the method was developed to quickly genotype SINE insertions in human populations, we reasoned that the technique could be modified to assess large numbers of SINE insertions among species.

Bats of the genus *Myotis* comprise one of the most species-rich mammalian clades, with over 100 species distributed worldwide (Simmons 2005). The availability of the *M. lucifugus* draft genome has led to extensive study of its TEs (Pritham and Feschotte 2007; Ray et al. 2007, 2008; Thomas et al. 2011; Pagán et al. 2012). Beyond the recent DNA transposon activity mentioned above, Ves SINEs have been active over the last 60 Myr in several bat families and have been shown to be phylogenetically informative (Borodulina and Kramerov 1999; Kawai et al. 2002; Pagán et al. 2012). Here, we describe a modified ME-Scan protocol to identify polymorphic SINE insertions in the genus *Myotis.* We identified over 796,000 Ves SINE insertions in six species of *Myotis* spanning a divergence period of 12 Myr (Stadelmann et al. 2007). Using approximately 85,000 phylogenetically informative insertions, we were able to construct a robust, TE insertion-based phylogeny. These results validate the use of the ME-Scan protocol for identifying TE polymorphisms in nonmodel taxa.

## Materials and Methods

### Taxon Selection

SINE libraries were generated for six New World (NW; *Myotis auriculus*, *M. dominicensis*, *M. lucifugus*, *M. occultus*, *M. simus*, *M. vivesi*) and one Old World (OW; *M. horsfieldii*) taxa (table 1) using an adaptation of the ME-Scan protocol (Witherspoon et al. 2010, 2013). Modifications were made to target the relatively young Ves3ML SINE subfamily, which is specific to vesper bats (Ray et al. 2015). Taxa were chosen based on their phylogenetic relationships as well as DNA availability, quality, and quantity. Field and museum identifications for each sample were validated by amplifying and sequencing a portion of the mitochondrial cytochrome *b* (*Cyt*b) gene using the methods of Stadelmann et al. (2007). Sequences were compared with records in GenBank before proceeding with library preparation steps.

**Table 1:** Voucher ID and Location of Specimens Used

Species Name	Voucher ID	Locality
New World		
*Myotis lucifugus*	MSB 40815	Oregon, United States
*Myotis auriculus*	MSB 40883	New Mexico, United States
*Myotis dominicensis*	TTU 31510	St. Joseph, Dominica
*Myotis simus*	TTU 46348	Huanuco, Peru
*Myotis occultus*	MSB 121995	New Mexico, United States
*Myotis vivesi*	MSB 42649	Sonora, Mexico
Old World		
*Myotis horsfieldii*	LSUM 4424	Pahang, Malaysia

Note.—MSB, Museum of Southwestern Biology, University of New Mexico; TTU, Natural Science Research Laboratory, Texas Tech University; LSUM, Museum of Natural Science, Louisiana State University.

### Library Preparation and Ves Enrichment

**Fig. 1.—:**
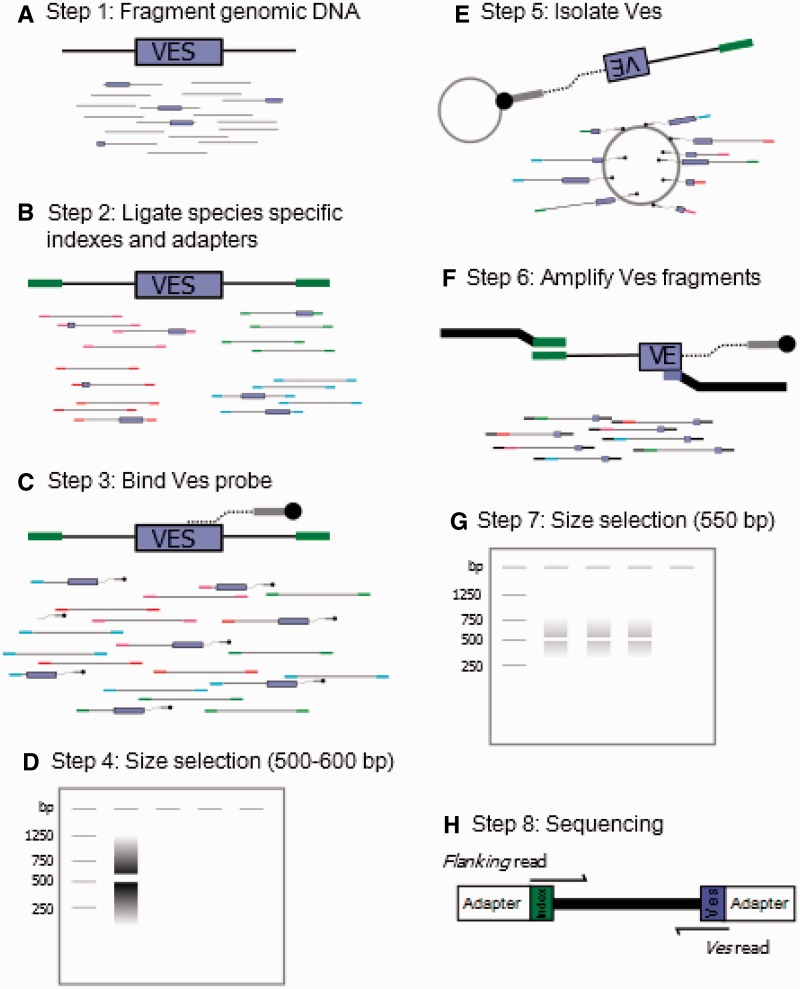
Preparation and sequencing of Ves enriched libraries. (*A*) gDNA is fragmented to an average size of 1 kb. (*B*) The fragmented, gDNA is end-repaired, and dA tailed before indexed adapters are added. (*C*) Individual libraries are pooled together and DNA fragments containing Ves are bound with a biotinylated Ves probe using a five-cycle PCR reaction. (*D*) The entire library is size-sorted along a gel. Fragments 500–600 bp in length are isolated. (*E*) The size-selected fragments are enriched for Ves by binding the Ves biotinylated probe to streptavidin-coated magnetic beads. (*F*) A final amplification of 15–20 cycles is used to amplify the library of Ves-enriched fragments for sequencing. (*G*) A final library of 550-bp Ves fragments is isolated through gel electrophoresis and purified for sequencing. (*H*) Fragments are sequenced so that one read contains the species-specific index plus DNA sequence up to 300–400 bp away from the Ves insertion (flanking read). The second read contains a portion of the Ves insertion plus approximately 80 bp of the Ves insertion site (*Ves* read).

A summary of the biochemical pipeline is shown in figure 1*A*–*H.* Genomic DNA (gDNA) was extracted from tissue samples using a standard phenol–chloroform/ethanol precipitation protocol. For each sample, 10 µg of gDNA was fragmented to an average size of 1 kb on a Covaris S220 Focused-ultrasonicator using the following parameters: Peak incident power, 105 W; duty factor, 5.0%; cycles per burst, 200; time, 40 s; temperature, 7 °C (fig. 1*A*). Fragmented gDNA was purified and concentrated using Qiagen QIAquick PCR purification columns (Qiagen, Germantown, MD) using the recommended protocol.

Fragmented gDNA was then prepared for Illumina sequencing using the End Repair, dA Tailing, and Adapter Ligation modules from the NEBNext DNA Library Prep Master Mix set (New England Biolabs, Ipswich, MA). During the adapter ligation step, custom, indexed adapters were ligated to each library for species identification (fig. 1*B*). Adapter and index sequences are presented in supplementary table S1, Supplementary Material online. After ligation, each indexed library was quantified using a NanoDrop ND-1000 spectrophotometer, and all seven libraries were pooled into a single library such that each species was equally represented. All subsequent steps were performed on this pool.

The combined pool of indexed gDNA fragments was enriched for Ves insertions by binding a biotinylated probe that is complementary to the consensus Ves3ML subfamily element (fig. 1*C*). The biotinylated probe targets a 20-bp region beginning at the 59th nucleotide of the Ves3ML consensus sequence. A five-cycle PCR was used to bind the Ves biotinylated probe to the gDNA fragments under the following thermal conditions: An initial denaturation of 98 °C for 30 s; five cycles of 98 °C for 10 s, 65 °C for 30 s, 72 °C for 30 s; and a final 72 °C 5 min extension. Reaction concentrations were as follows: 6 µl (∼150 ng) of pooled gDNA fragments, 1 µl (10 µM) Ves.btin.bp59 (biotinlyated probe), 1 µl (10 µM) Illumina P7 primer, 4 µl (5×) NEB Phusion HF buffer, 0.4 µl (10 mM) dNTPs, and 0.2 µl NEB Phusion Taq in a 20 µl reaction. Fragments between 500 and 600 bp were size selected through electrophoresis (fig. 1*D*) on a 15 cm, 2% agarose gel run at 80 V for 4 h and purified using the Qiagen gel extraction kit. Ves fragments bound by the biotinylated probe were magnetically isolated from gDNA fragments using streptavidin-coupled Dynabeads (fig. 1*E*), resulting in a library of Ves-enriched fragments between 500 and 600 bp, each containing a species-specific indexed adapter. The Ves fragments were then amplified for an additional 20 cycles (fig. 1*F*) with the standard Illumina P7 primer and another composite Ves + random sequence + Illumina P5 primer (supplementary table S1, Supplementary Material online). This Ves PCR primer was designed so that the first 20 bp were complementary to Ves, the next 3–6 bp alternated purines and pyrimidines, and the remaining 58 bp contained the P5 Illumina sequence. Amplification of the Ves fragments was done in three 25 µl reactions with 2 µl bead bound Ves fragments, 1 µl (10 µM) mixture of all 8 Ves composite PCR primer, 1 µl (10 µM) Illumina P7 primer, 5 µL (5 × ) NEB Phusion HF buffer, 0.5 µl (10 mM) dNTPs, and 0.4 µl NEB Phusion Taq using the previously mentioned cycling conditions. After amplification, bead bound Ves fragments were magnetically removed, and the PCR aliquot was electrophoresed at 40 V for 5 h on a 15 cm 2% agarose gel (fig. 1*G*). Ves fragments approximately 550 bp from all three amplified samples were purified from a gel excision using a Qiagen QiaQuick Gel Extraction column to produce the final sequencing library. Nanomolar concentration was calculated using a Qubit fluorometer. The final library of Ves fragments was sequenced on a single Illumina HiSeq 2000 through a commercial core lab (fig. 1*H*). The sequencing protocol allowed for 100 nt paired-end reads.

### Data Processing

Read pairs were expected to contain one read that spanned approximately 17–30 bp of the Ves insertion and up to 83 bp of gDNA, designated as the *Ves* read. The other read, the *Flanking* read, should consist of 100 bp of gDNA approximately 400 bp away from the insertion site. Both the *Ves* and *Flanking* reads were deconvoluted into species-specific files based on the 6-bp index identified in the *Flanking* read using Sabre (https://github.com/najoshi/sabre, last accessed June 8, 2015). Any reads where the sequence index did not perfectly match the template index were excluded from further analyses. After reads were deconvoluted, the *Ves* reads for each sequence pair were checked for complementarity to the 5′ 34 bp of the consensus Ves3ML using fastx_clipper from the FASTX-Toolkit (http://hannonlab.cshl.edu/fastx_toolkit/, last accessed June 8, 2015). Regions complementary to this portion of Ves3ML were clipped and any *Ves* reads lacking complementarity were discarded. In addition, any reads with an average Phred + 33 quality score less than 15 were removed. Processing the *Ves* and *Flanking* reads independently necessarily resulted in files where the number of sequences and sequence order were different. A custom perl script was used to reorganize the *Ves* and *Flanking* files so that orphaned reads were placed in a separate file and the complementarity of reads in both files was restored.

Paired reads were mapped to the 7 × *M. lucifugus* (Myoluc2.0: GCA_000147115.1) genome draft using BWA (Li and Durbin 2009) with the default options. Reads were initially mapped independently, then combined using Burrows-Wheeler Aligner (BWA) sampe based on an average insert size (‐a) of 400 bp. Using SAMtools (Li et al. 2009) read pairs were filtered so that only those mapping in the proper orientation (‐f 0 × 002) and within approximately 400 bp of its mate were kept. For these read pairs, the Ves insertion site was designated as the immediate nucleotide position on the 5′-end of the *Ves* read. All custom scripts used for data analysis are publically available (https://github.com/nealplatt/SineAnalysisTools, last accessed June 8, 2015).

### PCR Validation

To verify the ability of our method to capture polymorphic Ves insertions in *Myotis*, 20 loci were chosen for validation through PCR. Insertion sites to be tested were chosen at random after meeting the criteria listed. First, we allowed no other TE insertions (as annotated in the *M. lucifugus* genome) to fall within a ± 1-kb window of the Ves insertion site. Second, some insertions were recovered by only relatively few *Ves*/*Flanking* read pairs in one of the seven taxa, whereas others were supported by greater sequencing depth, or in multiple species. Only well-supported reads, that is, insertions identified using greater than ten read pairs, were included for PCR validation. Finally validated insertions could not be within 1 kb of distinct insertions identified in any of the other species. For those loci that met the requirements, the insertion position plus 500 bp of flanking sequence was extracted from the *M. lucifugus* genome. Using BatchPrimer3 (You et al. 2008), primers were designed to flank the expected Ves insertion site plus 100 bp on the 5′- and 3′-ends so that when amplified, the size of each locus would vary by approximately 200 bp based on the presence or absence of the Ves insertion (supplementary table S2, Supplementary Material online).

The predicted insertion sites were then amplified in seven *Myotis* taxa. For certain samples, the available DNA was exhausted during library preps. For these taxa, conspecific samples were used. PCR reaction conditions were as follows: 1.5 µl (10×) buffer, 0.7 µl *Redi*load loading dye, 1 µl Taq, 0.9 µl of MgCl_2_ (25 mM), 0.3 µl of each primer (10 µM), 0.3 µl of dNTPs (10 mM), 15 ng of gDNA, and water to 15 µl. Samples were amplified through an initial 94 °C denaturation period of 1 min, then 32 cycles of a 45 s 94 °C denaturation, 45 s at 52 °C annealing, and a 45 s 72 °C extension period, followed by a final 3 min 72 °C extension. Amplicons were visualized on a 15 cm, 1.5% agarose gel and electrophoresed at 140 V for 60 min. After visual inspection, a minimum of one sample exhibiting a band corresponding to the presence of a Ves insertion was purified using Qiagen QIAquick PCR purification columns. Samples were bidirectionally sequenced using internal primers binding to the 5′ and 3′ regions of the Ves element.

### Parsimony Analyses

A phylogeny for the specimens examined was generated using the Ves insertion profile for each taxon. Based on the estimated accuracy, discussed above, all insertions within 10 bp were assumed to be orthologous and coded as present (1) or absent (0). Precise excision of retrotransposon insertions is rare (van de Lagemaat et al. 2005; Ray et al. 2006) so the presence–absence data were analyzed under the assumption of Dollo’s principle (insertions are gained but never lost). Due to the failure of the *M. lucifugus* sequencing libraries, insertions of this species were coded as either present (1) or missing (?). A hypothetical ancestral taxon was created to serve as an artificial outgroup for phylogenetic analysis. As the presumed state of all loci is the absence of an insertion, all insertions were coded as absent in the putative ancestral taxon. Dollo parsimony analyses were conducted in PAUP* 4.0 (Swofford 2003) using equal weighting of all characters and tree-bisection reconnection with random addition (ten replicates) of taxa. Nodal support for the most parsimonious topology was estimated using 10,000 bootstrap replicates.

## Results

Sequencing the Ves libraries on a single Illumina HiSeq lane yielded over 103.5 million read pairs. All but approximately 297,400 read pairs were retained (i.e., binned into species-specific files based on the 6-bp index at the beginning of each *Flanking* read). Despite individually tagged gDNA libraries being pooled in equal proportions, the number of reads per taxon varied by more than 2.5 orders of magnitude, from approximately 5.8 thousand (*M. lucifugus*) to over 23.4 million (*M. auriculus*; table 2), though all taxa other than *M. lucifugus* had approximately 10 million or more read pairs. Because gDNA samples are indexed (fig. 1*B*) and pooled in equal proportions early in the library preparation process it likely that the *M. lucifugus* library preparation protocol failed prior to this point. Once the libraries were pooled, any failed downstream steps would have affected all taxa equally.

**Table 2:** Basic Read Counts for Each Taxon during the Data Analysis

	*Myotis auriculus*	*Myotis dominicensis*	*Myotis horsfieldii*	*Myotis lucifugus*	*Myotis occultus*	*Myotis simus*	*Myotis vivesi*
Total number of read pairs	23,422,455	18,459,538	17,806,029	5,790	20,257,386	9,802,564	13,496,084
Read pairs with expected Ves	23,380,075	18,417,016	17,765,391	5,770	20,218,366	9,766,555	13,465,245
Percent of total	99.82	99.77	99.77	99.65	99.81	99.63	99.77
Read pairs mapping to reference	18,385,217	14,570,546	10,557,215	3,841	17,457,290	6,398,914	10,810,003
Percent of total	78.49	78.93	59.29	66.34	86.18	65.28	80.1
Number of Ves identified	143,322	138,002	127,490	533	141,435	125,559	120,214
Average coverage per Ves	128.3×	105.6×	82.8×	7.2×	123.4×	51.0×	89.9×

Based on the design of the Ves PCR composite primer, approximately 30 bp of Ves sequence was expected at the beginning (5′) of each *Ves* read. The expected Ves sequence was identifiable in 99.6–99.8% of *Ves* reads from all retained read pairs. More than three-quarters (75.6%) of all read pairs mapped to the *M. lucifugus* genome, with success ranging from 59.3% (*M. horsfieldii*) to 86.2% (*M. occultus*; table 2).

Almost all mapped *Ves* reads from a single taxon within a ± 100-bp window fell very close to other reads. In total, approximately 99.5% of all reads were within ± 10 bp of each other (supplementary fig. S1, Supplementary Material online). Further, 96.7–98.7% of all reads within ± 100-bp window were supported by other reads at the same position (±0 bp). Based on the precision in read mapping determined a posteriori, reads within a ± 2-bp window were merged into the nucleotide position most commonly designated as the Ves insertion site. Insertion positions within each taxon were identified based on the position with the largest number of reads within any continuous stretch. For example, if *Ves* reads identified chr1:100-102 as potential insertion positions in *M. auriculus*, but chr1:100 was supported by 10 *Ves* reads, chr1:101 by 1 *Ves* read, and chr1:102 by 140 *Ves* reads, chr1:102 was designated as the true insertion position.

**Fig. 2.—:**
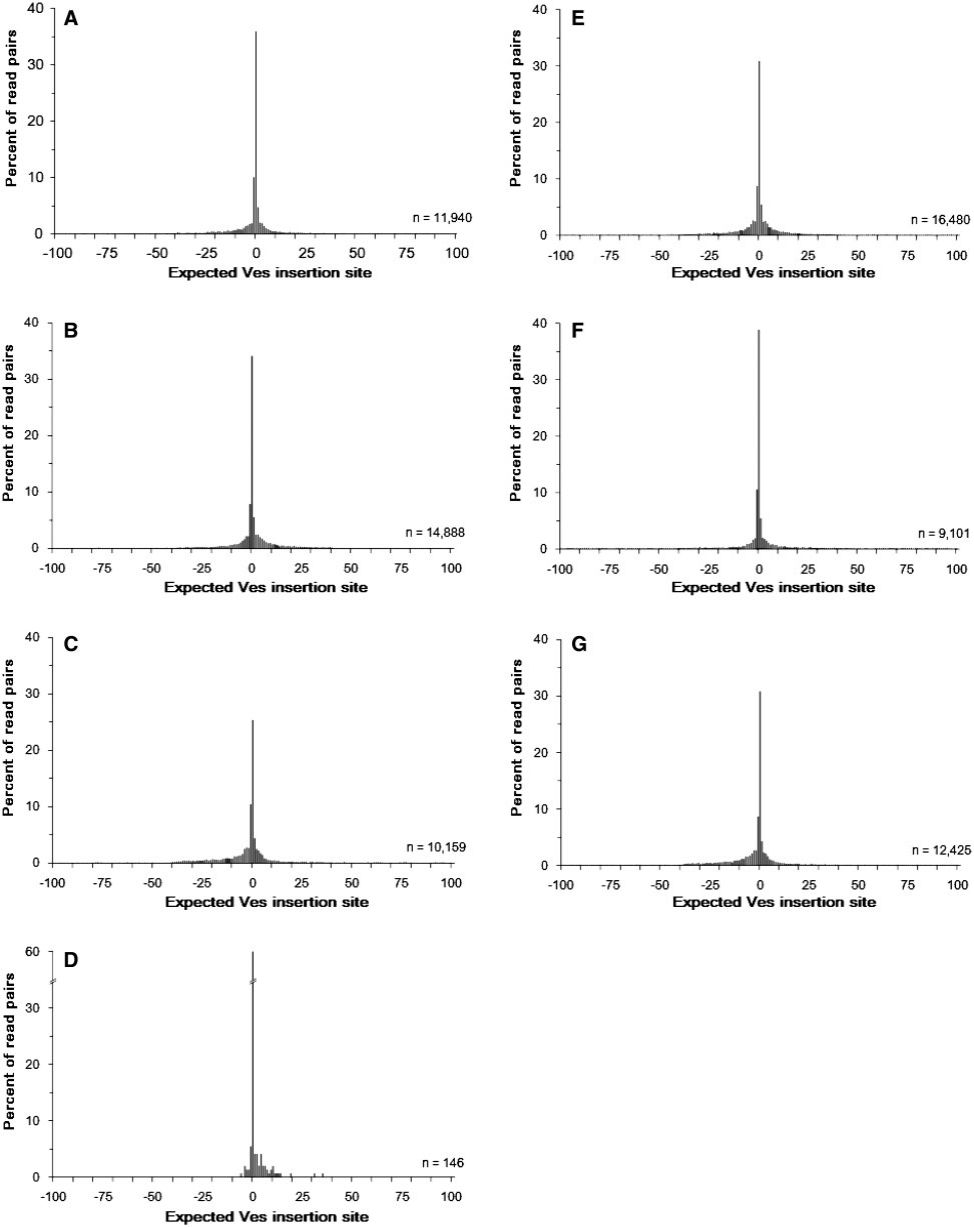
Accuracy of estimated Ves insertion sites shared among species. Estimated Ves insertion sites within each species ([*A*] *M. auriculus*, [*B*] *M. dominicensis*, [*C*] *M. horsfieldii*, [*D*] *M. lucifugus*, [*E*] *M. occultus*, [*F*] *M. simus*, [*G*] *M. vivesi*) were compared with the six other species examined. For estimated insertion sites that fell within ± 100 bp of each other between species, the distance was calculated. Orthologous insertions are expected to occur at the same position, whereas independent insertions would occur randomly.

After determining the estimated insertion position for each individual taxon, insertion positions between taxa were compared. Between 120,214 (*M. vivesi*) and 143,322 (*M. auriculus*) unique Ves insertions were identified, excluding *M. lucifugus,* which sequenced poorly (table 2). When insertions from different taxa were within 100 bp of each other, a majority were within 10 bp of each other (fig. 2). Insertions within this narrow window were assumed to be orthologous for downstream analysis.

**Fig. 3.—:**
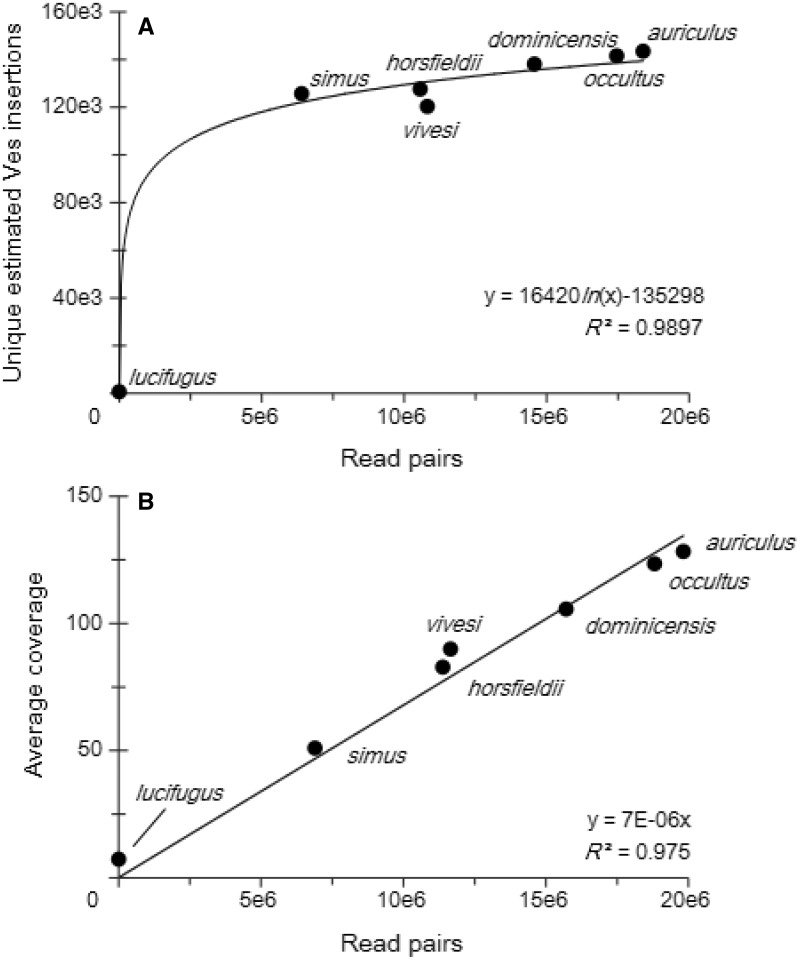
Sequence statistics per Ves insertion. (*A*) After approximately 5 million reads, the number of Ves insertions identified tended to stabilize so that more sequencing did not identify additional insertions, proportionally. (*B*) The average coverage of each Ves insertion is directly related to the number of read pairs.

On average, each Ves insertion was sequenced to 129.6 × coverage. Although *M. auriculus* was sequenced 2.5 × (13.6 million read pairs) more frequently than *M. simus*, the difference in number of Ves insertions identified was less than 17,800 insertions. Assuming these two taxa that diverged approximately 10 Ma (Stadelmann et al. 2007) have similar numbers of Ves insertions, this suggests that a saturation point is reached where more sequencing does not significantly increase the number of unique insertions discovered (fig. 3*A*). Our results indicate that after approximately 5 million read pairs, most Ves insertion sequences are represented. Additionally, when the number of reads pair is compared with the number of reads per Ves insertion, a strong linear relationship is recovered (*R*^2^ = 0.975; fig. 3*B*).

**Fig. 4.—:**
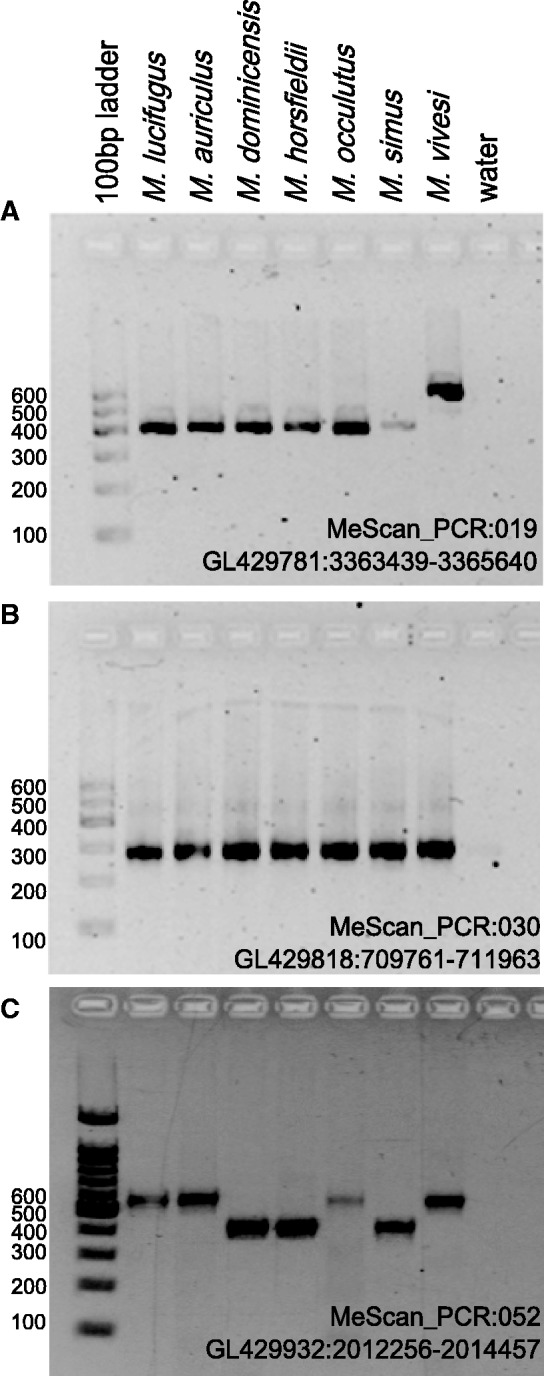
Results of Ves insertion panel on seven species of *Myotis* with three different loci. The names of each locus and its location in the *M. lucifugus* genome (Myoluc2.0: GCA_000147115.1) are given. Loci with a Ves insertion are expected to be approximately 200 bp larger than those lacking an insertion. Loci presented are examples of lineage specific (*A*), ancestral (*B*), and polymoprhic (*C*) insertion patterns. Based on the species analyzed here, only the polymorphic insertion locus is useful for phylogenetic analyses.

To verify the presence of Ves insertions identified by *Ves* reads, 42 loci were amplified in all seven taxa (fig. 4). Filled sites are expected to be approximately 200 bp larger than empty sites. Of the loci tested, 32 of 42 amplified in five or more samples, and 30 of the 32 primer pairs produced amplicons of predicted sizes confirming the expected insertion genotype. After verifying the increase in band size that is expected from a Ves insertion, loci were sequenced using internal Ves primers to confirm the approximately 200 bp insertion was indeed a Ves. For each locus, either one or two taxa were selected for sequencing to verify the presence of the Ves insertion as well as the expected flanking DNA sequence. In each case, the Ves sequence was found as expected.

**Fig. 5.—:**
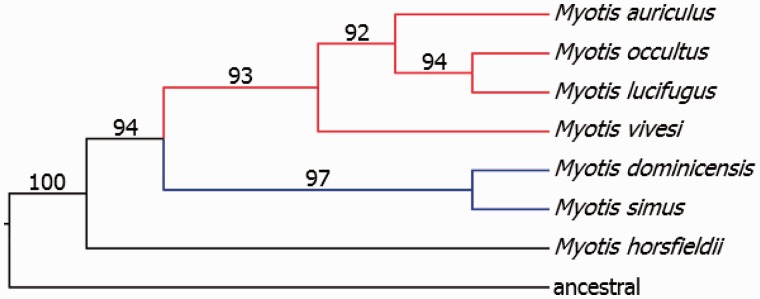
Phylogeny of *Myotis* based on Ves insertions. Dollo parsimony analyses of the Ves insertion data produced a single most parsimonious tree. Insertion data were coded as either present (1) or absent (0). Due to the failure of the *M. lucifugus* sequencing libraries, insertions of this species were coded as either present (1) or missing (?). Bootstrap percentages from 10,000 replicates are shown above the branches. Biogeographic distributions are designated by colored branches (blue, neotropical; red, nearctic). As the ancestral state of each Ves insertion is assumed to be its absence, a putative ancestral taxon was created where all insertions were coded as absent.

To recreate the phylogenetic relationships of available taxa, we used an unweighted Dollo parsimony analysis. After excluding derived Ves insertions, those present in only one species, 85,028 parsimony-informative characters were retained for analyses. Dollo parsimony resulted in a single most-parsimonious tree (fig. 5; length = 166,575 steps, consistency index = 0.518. rescaled consistency index = 0.166, retention index = 0.321, homoplasy index = 0.4820). Following 10,000 bootstrap replicates, all nodes received strong support.

Here, orthologous insertions in six taxa lacking reference genomes were identified by mapping *Ves* reads to the *M. lucifugus* reference genome. Though the presence of the *M. lucifugus* genome draft expedited these analyses, having a genome draft is not a prerequisite. For each insertion, more than 70 bp of gDNA from the *Ves* read and another 100–150 bp from the *Flanking* read are recovered. It is therefore possible to identify orthologous insertions through an all-versus-all comparison of similarity in these genomic regions across all taxa examined. Thus, all that is necessary to generate a SINE-based phylogeny for taxa lacking closely related reference genomes is a minimal knowledge of the SINE family to be targeted for probe construction. Because SINEs are so abundant in eukaryotic genomes this information could be obtained by low coverage (less than 0.1 × ) genome sequencing at costs of a few hundred dollars (Pagán et al. 2012; Ray et al. forthcoming).

## Discussion

Our goal was to identify polymorphic Ves insertions in *Myotis* species for which no genome is available by relying on the closely related *M. lucifugus* genome as a reference. To do this, we used the ME-Scan (Witherspoon et al. 2010, 2013) protocol where biotinylated probes are used to enrich high-throughput DNA sequencing libraries for SINE containing DNA fragments. In the current implementation, the biotinylated probes were used to target Ves SINEs across seven species of *Myotis.* After sequencing, insertion positions were identified and analyzed bioinformatically to validate the protocol. The mapping ability and high Ves recovery rate in *Myotis*, combined with high *Alu* recovery rates (94.3%) in humans (Witherspoon et al. 2010), indicate that the ME-Scan method is highly efficient for sequencing large numbers of SINE insertions under a range of conditions and should be extendable to SINE families in other taxa.

To use the Ves insertions identified in the sequencing and computational steps above, the insertion position must be identified with a high degree of precision. This is particularly important for shared or polymorphic insertions whose inferred presence or absence could be useful for phylogenetic analysis. A direct relationship exists between insertion site spacing and our ability to identify unique insertion events. Two SINE insertions separated by 100–1,000 bp are easily identifiable as unique insertions. However, when one reduces that distance to 1–10 bp, their uniqueness is more difficult to determine. Insertions within a narrow window could be homoplastic or could be caused by imprecise mapping of multiple reads from the same insertion. To address this question, the distance to the nearest neighboring insertion for each insertion position was calculated in all seven taxa. Although two insertions separated by 10 bp in different species could be due to imprecise read mapping, sequencing errors, or (near) homoplasy, insertions separated by 10 bp from the same individual are almost certainly due to imprecise mapping or sequencing errors. When closest neighbors were calculated within each species, we found that almost all *Ves* reads supported insertion sites within a very narrow window (supplementary fig. S1, Supplementary Material online).

Once insertion positions had been identified in each species, these positions were compared among species. Similar to the within-species results, insertion positions among species tended to be identified with a high degree of accuracy within a ± 10-bp window (fig. 2). Though precise parallel insertions cannot be completely ruled out, mapping accuracy is strong evidence that identified insertion positions within a narrow window are likely shared, orthologous insertions acquired from the common ancestor. On the other hand, even if precise parallel insertion events cannot be ruled out, these events are expected to occur at such a low frequency that they would not be expected to substantially impact our results (Ray et al. 2006). Combined, these two semi-independent lines of evidence (precision within species and accuracy among species) suggest that reads mapping within a narrow window are very likely to have originated from the same ancestral insertion, an assumption that is important for phylogenetic and population genetic inquiries.

The results provide strong evidence that there are approximately 142,000 Ves insertions recoverable using the ME-Scan method and our probe design. There are 544,807 Ves insertions divided among four subfamilies (Ves2_ML = 100,744; Ves2B_ML = 121,261; Ves3_ML = 278,544; Ves4_ML = 44,258) identified in the *M. lucifugus* genome (http://repeatmasker.org/species/myoLuc.html, last accessed June 8, 2015). Our probes were designed to bind to positions within Ves that are specific to the Ves3_ML subfamily, yet we are able to recover only approximately 50% of the expected number of insertions. To determine whether our probe design or number of reads was the limiting factor in the number of Ves recovered, the Ves portion of the biotinylated probe and Ves PCR composite primers were queried against the *M. lucifugus* genome. To identify a capturable binding site, all portions of the genome complementary to the Ves portion of our biotinlyated probe were searched for Ves PCR composite binding sites within a ± 50-bp region. Using these criteria, a maximum of 164,689 potentially capturable Ves sites exist within *M. lucifugus.* If this number is valid, then we captured almost 70% of potential sites, and are likely sequencing Ves fragments to saturation. Further optimization of probe design and hybridization protocols will increase the capture rate. These observations provide confidence in the ability to identify Ves insertions in taxa lacking a reference genome.

### Phylogenetics of *Myotis* Using Polymorphic Insertions

The presence or absence of Ves insertions has been previously shown to be informative for vespertiolionid phylogenetics (Kawai et al. 2002) and our results support this conclusion. Our unweighted Dollo parsimony analysis of more than 85,000 informative characters resulted in a single most-parsimonious tree (fig. 5) with significant bootstrap support at all nodes. The topology recovered using Ves insertions is similar to that of the mitochondrial *Cyt*b gene, differing only in placement of *M. vivesi* (Ruedi and Mayer 2001; Stadelmann et al. 2007). *Cyt*b analyses place *M. vivesi* among the neotropical *Myotis* species (*M. dominicensis* and *M. simus*). The Ves insertion analysis places *M. vivesi* basal to the other nearctic species. It is difficult to draw specific conclusions based on the limited taxon sampling presented herein, but the Ves insertion phylogeny recovers monophyletic clades of Neotropic and Nearctic species, indicating a single invasion into each of these regions. This is in contrast to a phylogeny based on combined nuclear and mitochondrial DNA that suggests multiple neotropical invasions (Stadelmann et al. 2007). Inclusion of additional species and replicates of the included taxa should resolve these conflicts. However, our results strongly suggest that this method could be a valuable addition to the phylogeny inference toolkit.

The method is not without problems, however. Because the capture protocol relies on probes binding to Ves, sequence reads only indicate the presence of a Ves insertion, the data cannot confirm the absence of Ves insertions. However, our results demonstrate that all captured Ves insertions were sequenced at an average depth of approximately 130 × . Thus, it is very likely that the lack of a *Ves* read represents an empty site but other nontrivial methods would be necessary to confirm the lack of an insertion at any particular locus. In the past, this has been accomplished through PCR amplification and electrophoresis of the locus. Although this is possible for dozens or even hundreds of loci, this task quickly becomes overwhelming when tens of thousands of loci are to be examined. The ME-Scan protocol could conceivably be modified to verify insertion absence. For example, after identifying polymorphic loci through the methods used herein, one could build probes to bind to genomic flanking sequences of each insertion and sequence into the Ves locus. For taxa that lacked the Ves insertion the sequence would read into the genomic flanking regions, whereas Ves containing loci would contain Ves sequence. This method would however require generating thousands of unique probes, more than 85,000 in this case.

Parallel insertions could lead to the opposite results, a false positive where two independent insertion events occurred at the same locus. However, it is thought that indistinguishable parallel insertions are rare (Ray et al. 2006; Han et al. 2011). Our data indicate that it is possible to distinguish near-parallel insertions as long as they occur more than 25 bp from each other (fig. 2), or are in opposing orientations. For example, as the biotinylated probe binds to the 5′ region of the Ves insertion all fragments contain approximately 30 bp of Ves and the upstream genomic flanking sequence. If two insertions occurred independently at the exact some locus and were in opposite orientations, they would be distinguishable. It is only in the case of independent insertions occurring within a very narrow window (<25 bp) and in the same orientation, that parallel insertions become a problem, a problem that is not unique to the ME-Scan protocol. Any insertion-based data set would struggle to discern parallel insertions. Because false negatives (described above) and false positives (described here) are possible, individual loci may contain some phylogenetic ambiguity, however, when distributed over thousands of loci most of this ambiguity is removed.

## Conclusions

Retrotransposons are being used with increasing frequency as genetic markers for identification of taxa ranging from individuals to orders (Ryan and Dugaiczyk 1989; Murata et al. 1993; Schmitz et al. 2001) (for additional examples, see Ray et al. 2005; Xing et al. 2005, 2007; Herke et al. 2007; Suh et al. 2011, 2014; Haddrath and Baker 2012; McLain et al. 2012; Meyer et al. 2012). In the past, identifying polymorphic SINE loci required expensive and time-consuming laboratory techniques such as modified bubble-PCR (Roy et al. 1999; Ray and Batzer 2005; Xing et al. 2005) or inverse PCR from circularized DNA fragments (Suh et al. 2012). While effective, these techniques yielded only a few hundred useful loci at best. As new sequencing technologies have become available, the ability to identify SINE insertions on a large scale has become feasible. Further, as the number of reference genomes increases, many nonmodel taxa will have closely related genomes available for comparative bioinformatic analyses.

The ME-Scan protocol (Witherspoon et al. 2010, 2013) was modified to target Ves elements in seven species of *Myotis.* We show that large numbers of insertions can be captured in taxa separated by up to 12 Myr (Stadelmann et al. 2007), and that insertion positions can be identified to a narrow window within and among species, all without relying on a reference genome. In *Myotis*, we identified over 796,000 SINE insertions in seven taxa. On average, each insertion was supported by a large number of reads (129.6×), and each species (excluding *M. lucifugus*) recovered similar numbers of Ves insertions. Though the number of reads varied greatly among taxa, most Ves were recovered after 5 million sequence reads. Future work will increase the number of taxa to generate a phylogenetically informative data set with broader sampling.

## Supplementary Material

Supplementary tables S1 and S2 and figure S1 are available at *Genome Biology and Evolution* online (http://www.gbe.oxfordjournals.org/).
